# Honey-Related Treatment Strategies in Dry Eye Disease

**DOI:** 10.3390/ph16050762

**Published:** 2023-05-18

**Authors:** Julia Prinz, Nicola Maffulli, Matthias Fuest, Peter Walter, Frank Hildebrand, Filippo Migliorini

**Affiliations:** 1 RWTH University Hospital of Aachen, 52074 Aachen, Germany; 2 Department of Medicine, Surgery and Dentistry, University of Salerno, 84081 Salerno, Italy; 3 Barts and The London School of Medicine and Dentistry, Mile End Hospital, Queen Mary University of London, 275 Bancroft Road, London E1 4DG, UK; 4 School of Pharmacy and Bioengineering, Keele University Faculty of Medicine, Thornburrow Drive, Stoke on Trent ST4 7QB, UK; 5 Department of Orthopaedics and Trauma Surgery, Academic Hospital of Bolzano (SABES-ASDAA), 39100 Bolzano, Italy

**Keywords:** dry eye disease, xerophthalmus, keratoconjunctivitis sicca, honey, Manuka, Royal Jelly

## Abstract

This systematic review and meta-analysis investigated whether honey-related treatment strategies improve the signs and symptoms of patients with dry eye disease (DED). In March 2023, the following databases were accessed for clinical trials investigating the efficacy of honey-related treatment strategies in DED: PubMed, Web of Science, Google Scholar, and EMBASE. The following data were extracted at baseline and at the last follow-up: Ocular Surface Disease Index, tear breakup time, Schirmer I test, and corneal staining. Data from 323 patients were retrieved (53.3% female, mean age 40.6 ± 18.1 years). The mean follow-up was 7.0 ± 4.2 weeks. All the endpoints of interest significantly improved from baseline to the last follow-up: tear breakup time (*p* = 0.01), Ocular Surface Disease Index (*p* < 0.0001), Schirmer I test (*p* = 0.0001), and corneal staining (*p* < 0.0001). No difference was found in tear breakup time (*p* = 0.3), Ocular Surface Disease Index (*p* = 0.4), Schirmer I test (*p* = 0.3), and corneal staining (*p* = 0.3) between the honey-related treatment strategies and the control groups. According to our main results, honey-related treatment strategies are effective and feasible to improve symptoms and signs of DED.

## 1. Introduction

Dry eye disease (DED) is a common ocular condition with a prevalence rate of up to 74% [1,2,3,4,5]. The aetiology of DED is multifactorial [2]. Inflammatory or environmental conditions, such as allergens, contact lens wear, cigarette smoke, previous eye surgery, neurotrophic deficiency, exposure to pollutants, ultraviolet radiation, hormonal imbalance—especially in perimenopausal women—and oxidative stress are implicated in DED [6,7,8]. Additionally, tear film hyperosmolarity plays an important role in the pathogenesis of DED: damage of the corneal epithelium leads to cell death by apoptosis, followed by a loss of goblet cells and mucin expression, increasing the presence of inflammatory mediators, such as tumour necrosis factor α, interleukin 6, and matrix metallopeptidase 9 [9,10]. Symptoms associated with DED include foreign body sensation, blurred vision, pain, and photophobia [6,11]. Aqueous-deficient and evaporative DED can be distinguished, while numerous patients show signs of both subtypes [1]. Current treatment options mainly comprise artificial tears, lifestyle changes, topical steroids or cyclosporine, lacrimal punctal occlusion, and oral omega-3 fatty acids [12,13,14,15,16]. Though improving both symptoms and clinical findings, artificial tears do not treat the inflammation processes underlying DED [17]. Topical steroids have anti-inflammatory properties, and their efficacy in improving the signs and symptoms of DED have been demonstrated before [16,18]. However, their long-term use is not recommended, as ocular side effects such as the development of cataract or secondary glaucoma might occur [19,20]. Further anti-inflammatory treatment strategies for patients with DED include cyclosporine and lifitegrast [21]. Recently, the efficacy of naturally occurring anti-inflammatory agents as treatment strategies for patients with DED has been investigated [21]. Altogether, the interest in alternative treatment approaches for DED is growing [21].

Honey has been used in the management of ophthalmic diseases for thousands of years [22]. It mainly consists of carbohydrates, water, organic acids, proteins, amino acids, minerals, vitamins, enzymes, flavonoids, and antimicrobial components, such as hydrogen peroxide, sugar, and antimicrobial peptides [23,24]. The flavonoids, including pinocembrin, quercetin, chrysin, and phenolic acids might be accountable for the anti-bacterial, anti-oxidant, anti-inflammatory, immunomodulatory and analgesic properties of honey [25]. Additionally, the anti-bacterial, anti-oxidant, and anti-fungal effects of honey have been attributed to the anti-microbial effects of glucose oxidase, and a high osmolarity which might inhibit bacterial growth [22,24,26]. More than 300 varieties of honey exist, depending on the heterogeneity of plants and environmental conditions [23]. Manuka honey contains a proprietary mix of honey from the Australian and New Zealand *Leptospermum* species (known as Manuka) [27]. Its anti-bacterial efficacy includes activity against methicillin-resistant *Staphylococcus aureus* and *Pseudomonas aeruginosa* [28]. Royal Jelly is mainly secreted by the worker honeybees (*Apis mellifera*) and is required for the nutrition of the queen honeybee [29]. It is secreted from the hypopharyngeal and mandibular glands of the worker bees [29]. In general, medical-grade honey is free of toxic contaminants after sterilisation by gamma irradiation, according to standard medical regulations [30]. The efficacy of honey in the treatment of ophthalmic diseases such as chemical and thermal burns, corneal bacterial ulcers, postoperative corneal oedema, bullous keratopathy, neurotrophic keratitis, vernal keratoconjunctivitis, and catarrhal keratoconjunctivitis has been demonstrated by previous studies [22,31,32,33,34]. It has been attributed to the capacity of honey to stimulate immune cells and promote reepithelialisation, as well as angiogenesis [35].

The present study investigated whether honey supplementation improves the clinical signs and symptoms in patients with DED at the last follow-up compared to baseline. Moreover, a meta-analysis comparing honey-derived therapies vs. placebo or artificial tears was conducted.

## 2. Results

### 2.1. Study Selection

**Figure 1:**
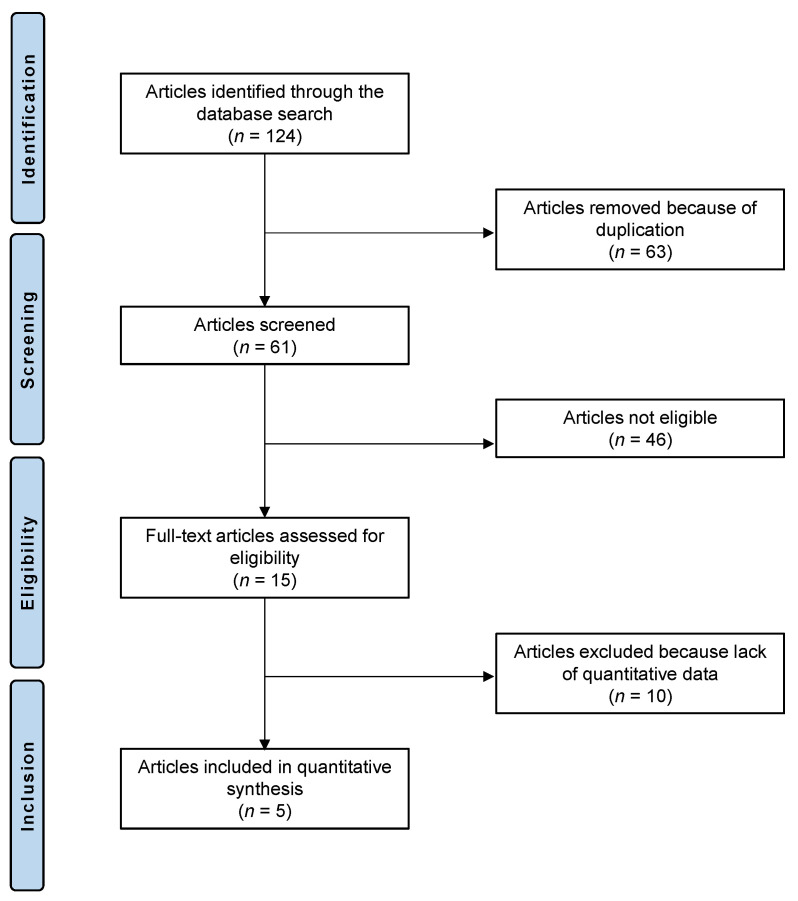
PRISMA flow chart of the literature search.

The eligibility criteria are described in detail in paragraph 4.1. The literature search resulted in 124 randomized clinical trials which evaluated the efficacy of topical or systemic honey application in patients with DED. Of them, 63 were excluded because of duplication. Another 46 articles were excluded because they did not match the eligibility criteria. Ten further studies did not report quantitative data under the endpoints of interest and were therefore excluded from further analysis. Finally, five randomized clinical trials were eligible for the final analysis. The flow chart of the literature search is shown in Figure 1.

### 2.2. Study Risk of Bias Assessment

**Figure 2:**
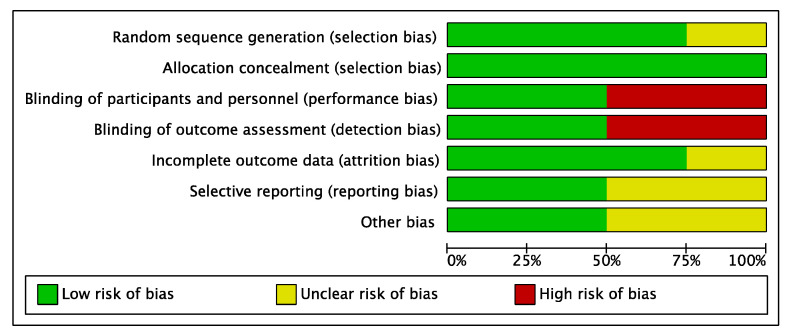
Methodological quality assessment.

Given the randomized design of the patient allocation in all included studies, the risk of selection bias was low. A moderate risk of detection bias was evidenced. Additionally, the performance bias was considered low. The risk of attrition bias was low, and the risk of reporting and other biases was moderate. Overall, the risk of bias graph evidenced a low risk of publication bias in the included studies. The results of the methodological quality assessment for the selection bias, performance bias, detection bias, attrition bias, reporting bias, and other biases for the included studies are shown in Figure 2.

### 2.3. Risk of Publication Bias

**Figure 3:**
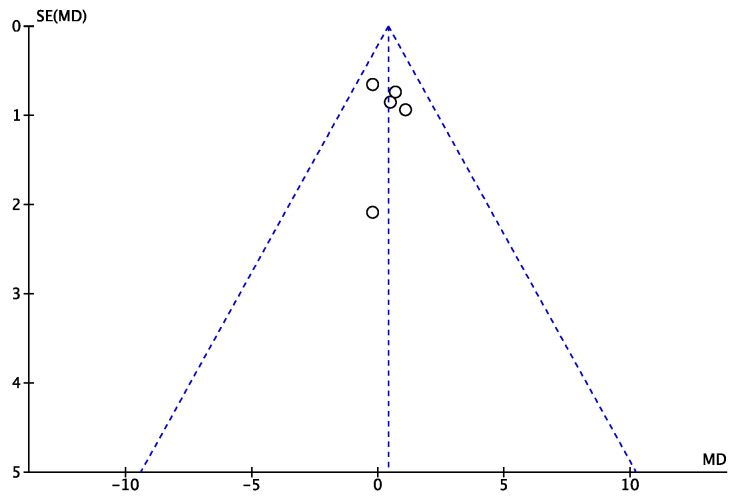
Funnel plot to evaluate the risk of publication bias.

The funnel plot was performed to evaluate the risk of publication bias of the present study. All the effects were located within the shape of acceptability, and they demonstrated a symmetrical disposition. These features of the plot indicate a low risk of publication bias (Figure 3).

### 2.4. Study Characteristics and Results of Individual Studies

Data from 323 patients were retrieved from the included studies. The study generalities and patient characteristics of the included studies are shown in greater detail in Table 1.

### 2.5. Efficacy of Honey-Related Treatment Strategies

All the endpoints of interest significantly changed from baseline to the last follow-up: At the last follow-up, the tear breakup time was significantly increased (+1.1 s; *p* = 0.01), the Ocular Surface Disease Index score was significantly reduced (−12.8 points; *p* < 0.0001), the Schirmer I test was significantly increased (+1.8 mm; *p* = 0.0001), and the corneal staining score was significantly reduced (−1.2 points; *p* < 0.0001) compared to baseline. These results are shown in greater detail in Table 2.

### 2.6. Honey-Related Treatment Strategies Compared to Other Treatments

**Figure 4:**
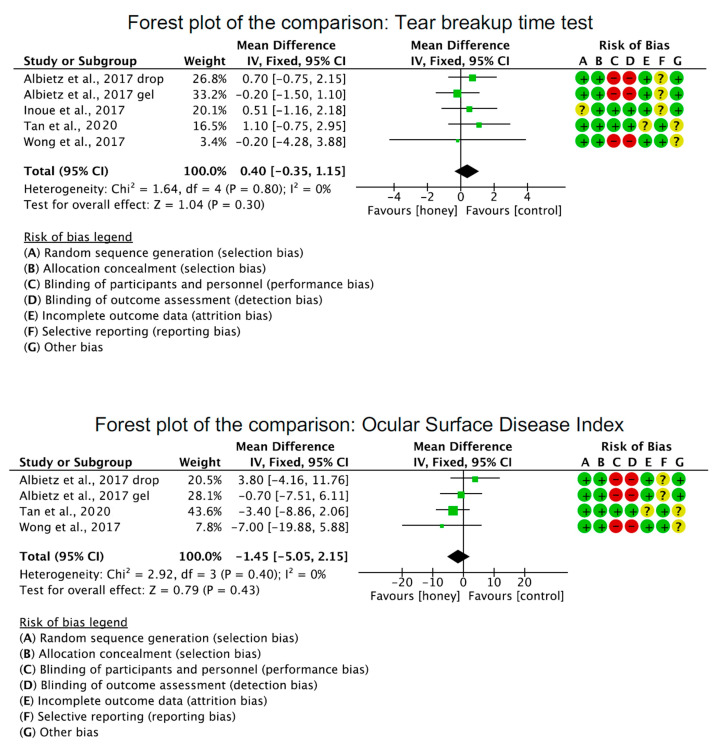

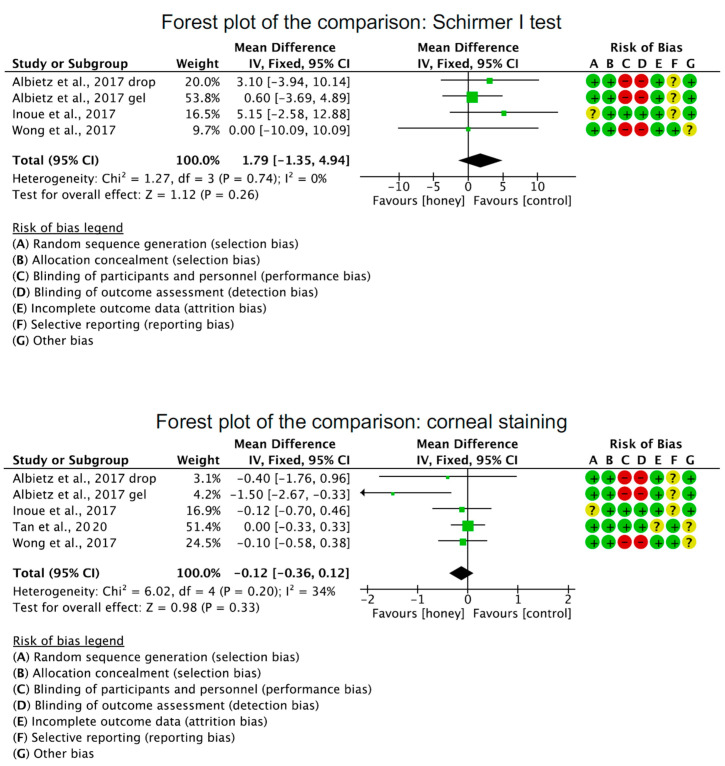
Results of the meta-analysis [27,29,36,38].

No significant differences were found in terms of the tear breakup time (*p* = 0.3), the Ocular Surface Disease Index (*p* = 0.4), the Schirmer I test (*p* = 0.3), and corneal staining (*p* = 0.3) between the honey-related treatment and the control group (Figure 4).

## 3. Discussion

According to the main findings of the present study, topical or systemic honey-related treatment strategies led to a significant increase in the tear breakup time and the Schirmer I test at the last follow-up compared to baseline in patients with DED. At the last follow-up, the Ocular Surface Disease Index score and corneal staining were significantly lower compared to the baseline in the honey-related treatment group. No differences in the tear breakup time, Schirmer I test, corneal staining, or Ocular Surface Disease Index scores between the honey-related treatment group and the control groups were identified. The proportion of females (53%) and age distribution in this study agree with previous publications [39,40].

Studies using both Manuka honey and Royal Jelly as a treatment in patients with DED were included in the present systematic review and meta-analysis. Manuka honey is a monofloral honey originating from the Manuka tree (*Leptospermum* sp.) [41]. Royal Jelly has been shown to have anti-bacterial, anti-inflammatory, and anti-fungal properties, and has been used to treat a variety of disorders in humans, including ocular diseases [42], as well as diabetes, or Alzheimer’s disease [43]. Its exact mechanism of action is not completely understood. However, Royal Jelly has been reported to stimulate the mobilization of calcium ions via muscarinic signal transduction pathways in the lacrimal glands [21].

Recent studies showed that oxidative stress damages the ocular surface and plays an important role in DED [44]. Markers of oxidative stress, such as lipid peroxidase, myeloperoxidase, nitric oxide synthase, and reactive oxygen species were previously found in the tears, conjunctival cells, and conjunctival biopsies of patients suffering from DED [45]. Oxidative stress occurs when the balance between the level of reactive oxygen species and the level of protective enzymes is disrupted [46]. The ocular surface is constantly exposed to the burden of free radical stress caused by ultraviolet radiation and environmental pollution [8]. Honey has been demonstrated to show anti-oxidative properties as it causes free radicals to neutralize [22,47]. These anti-oxidant properties are mainly attributable to the flavonoids, carotenoids, and phenolic acids which are present in honey [48]. Flavonoids are a group of plant secondary metabolites which are known for their anti-oxidative, anti-inflammatory, anti-carcinogenic, and anti-mutagenic properties [49]. The phenolic acids contained in honey are capable of chelating ferrous ions and scavenging hydrogen peroxide [50]. Generally, anti-oxidants also have an anti-inflammatory effect given that oxygen free radicals are involved in several inflammatory conditions [51,52].

At the last follow-up, the tear breakup time was increased in the honey group compared to the control group. Recently, honey has been reported to promote the secretion of tears by the lacrimal gland [47]. A previous study demonstrated that orally supplied Royal Jelly promoted tear secretion in blink-suppressed dry eye animal models [47]. Furthermore, an increase in adenosine triphosphate (ATP) and mitochondrial function by modulation of the calcium signalling pathway has been demonstrated after oral administration of Royal Jelly, suggesting a restoration in the lacrimal production by the gland cells [47].

The efficacy of Manuka honey in reducing the bacterial colonization of the lid margin has been described in previous studies [36]. Moreover, a significant reduction in inflammatory markers after honey supplementation, such as matrix metallopeptidase 9, has been presumed [36]. DED from blepharitis is mainly attributed to bacterial side products rather than the bacteria themselves [53]. Thus, honey might reduce the susceptibility of DED patients to bacterial conjunctivitis and relieve the symptoms of DED by reducing the production of bacterial side products, e.g., bacterial lipases [54]. These bacterial lipases are believed to hydrolyse the lipids of the meibomian glands, thereby releasing free fatty acids which might destabilize the tear film and have toxic effects on the corneal epithelium [55]. Tear film hyperosmolarity has been shown to stimulate multiple inflammatory reactions on the ocular surface, which result in apoptotic cell death of conjunctival epithelium and goblet cells [28,56]. Decreasing goblet cell densities result in increased tear film evaporation, which is one important mechanism of DED [28,56]. However, the exact underlying mechanisms are still unclear.

Whereas no significant change in corneal staining was observed in the study by Tan et al. [38], Albietz and Schmid reported a significant reduction in interpalpebral corneal and conjunctival staining after Manuka honey [36]. These divergent results might be attributable to the different underlying grading scales: Tan et al. [38] graded the extent of staining for each quadrant using the Centre for Contact Lens Research Unit (CCLRU) grading scale [57] and averaged the results for the quadrants to obtain an overall score per eye [38], whereas Albietz et al. [36] used the Oxford Scheme [58] in their study [36]. Moreover, differences in baseline characteristics might explain the inhomogeneous results between the studies. In this regard, patients in the study by Albietz et al. [36] had a numerically higher Ocular Surface Disease Index score at baseline compared to patients in the study by Tan et al. [38] (38.2 vs. 33.7, respectively). Previously, honey has been demonstrated to stimulate angiogenesis, granulation, and epithelization [59]. In this context, it has been suggested that honey might stimulate cytokine production (e.g., tumour necrosis factor α or interleukin 6) from human monocytes [35]. However, the exact components of honey responsible for this effect and the precise mechanism of action are not yet fully understood [60]. Another suggestion on the efficacy of honey included the presence of microorganisms in honey, such as aerobic and anaerobic bacteria [61]. Hypothetically, the presence of microorganisms in honey explains its stimulation of immune cells [60,61]. However, recently, Tonks et al. identified a 5.8 kDA component of Manuka honey that is responsible for stimulating inflammatory responses including cytokine production in human monocytes via the Toll-like receptor 4 [60].

Some between-studies heterogeneities should be considered. Topical honey as a treatment for DED has been demonstrated to improve ocular comfort after 2 [27] to 13 weeks of follow-up [37]. Wong et al. investigated the effect of Manuka (*Leptospermum* sp.) honey eye drops in 24 patients with contact-lens-related DED [27]. They reported improvements in subjective symptomology as measured by the Ocular Surface Disease Index score in symptomatic patients. However, no improvements in objective signs, such as the Schirmer I test or tear breakup time, were demonstrated, which was attributed to the short follow-up period of 2 weeks by the authors [27]. Albietz et al. evaluated the efficacy of Manuka (*Leptospermum* sp.) combined with conventional therapy for DED, consisting of warm compresses, artificial tears, and lid massage, involving 114 patients. The authors reported significant improvement in corneal staining and meibum quality after therapy with Manuka. In addition, treatment with Manuka reduced the need for artificial tears [36]. Tan et al. [38] compared the efficacy of Manuka honey to artificial tears. The study included 46 patients with DED. After a follow-up of 4 weeks, patients treated with Manuka honey had significantly lower Ocular Surface Disease Index scores compared to the control group. Additionally, the authors reported a slight but not statistically significant increase in tear breakup time in the Manuka honey group at 4 weeks follow-up compared to baseline [38]. In a recent study by Craig et al. [37], the efficacy of an eye cream consisting of Manuka honey microemulsion on 53 patients with DED and blepharitis was evaluated [37]. After 3 months, topical Manuka honey resulted in significant improvements in subjective symptomology and tear breakup time [37]. Inoue et al. investigated the efficacy of Royal Jelly honey administration in 43 Japanese patients compared to a placebo for 8 weeks [29]. The authors found that the tear volume significantly increased following treatment with Royal Jelly honey. In patients with a baseline Schirmer I test value of ≤10 mm, a significant increase compared to baseline tear volume and also compared to the placebo group was witnessed [29]. No severe treatment-related adverse effects were reported in the included studies [27,36,37,38,62]. Minor adverse effects related to the topical honey eye drop or eye cream instillation included temporary stinging and discomfort in two of the included studies [36,37], while the other studies did not report any adverse events attributable to the topical honey treatment. To date, no further ongoing studies evaluating the efficacy of honey for DED are registered in the U.S. National Library of Medicine.

This study has several limitations. The limited study size was the most important limitation of the present study. Given the limited quantitative data available for inclusion, it was not possible to analyse different types of honey-related treatment strategies, such as Manuka honey or Royal Jelly, separately. Future comparative randomized controlled trials are warranted to investigate which type of honey might be most effective in patients with DED. The treatment protocols within the honey group were heterogeneous, including topical and oral honey administration and different honey types. Furthermore, different honey types which are derived from different plants might vary significantly in their composition and their anti-bacterial and anti-oxidant properties [63]. The control group was also heterogeneous: Conventional lubricant eye drops (Novartis International AG, Fort Worth, TX, USA) were used as a treatment in the control group by Wong et al. [27] and Tan et al. [38], and adjunctive conventional therapy including warm compresses, lid massage, and lubricants by Albietz et al. [36]. In the study by Inoue et al., patients in the control group received placebo tablets [29]. Craig et al. treated only one eye of their patients with Manuka honey microemulsion and left the second eye of their patients untreated as a control eye [37]. The heterogeneous length of the follow-up might also limit the reliability of our results. Different inclusion and exclusion criteria of the different studies were not accounted for. Future level I evidence studies should be undertaken to overcome current obstacles to clinical translation and study the role of topical honey treatment in DED more extensively. Moreover, future studies should focus on which cohort of DED patients can benefit from topical or systemic honey treatment compared to other treatment options for DED, such as artificial tears or anti-inflammatory agents.

## 4. Materials and Methods

### 4.1. Eligibility Criteria

All the clinical trials which investigated the efficacy of topical or oral application of honey-related treatment strategies for DED were accessed. According to the authors language capabilities, articles in English, German, Italian, French, and Spanish were eligible. According to the Oxford Centre of Evidence-Based Medicine [64], only level I evidence was considered. Reviews, opinions, letters, and editorials were not considered. Animals, in vitro, biomechanical, computational, and cadaveric studies were also not eligible. Only studies investigating patients affected by clinically manifest DED were eligible. Studies including patients with Sjögren’s syndrome, graft vs. host disease, or Stevens–Johnson syndrome-related severe DED were not considered. Studies investigating the efficacy of honey-related treatment strategies in patients receiving punctal occlusion procedures were excluded. All types of honey-related treatment strategies as a treatment of DED were considered eligible. Studies combining honey-related treatment strategies with other treatments, except for conventional therapy including artificial tears, were excluded. Only studies which reported quantitative data under the endpoints of interest were eligible.

### 4.2. Search Strategy

This meta-analysis systematic review was conducted according to the Preferred Reporting Items for Systematic Reviews and Meta-Analyses: the 2020 PRISMA statement [65]. The PICO algorithm was preliminary established:

- P (Population): patients with DED;
- I (Intervention): Honey-related treatment strategies, including Manuka honey, Royal Jelly;
- C (Comparison): improvement at the last follow-up and compared with placebo or control group;
- O (Outcomes): Ocular Surface Disease Index; Tear breakup time test; Schirmer I test, corneal staining, adverse events.

In March 2023, the following databases were accessed: PubMed, Web of Science, Google Scholar, and Embase. No time constraints were used for the search. The following keywords were used in combination: dry eye disease, xerophthalmus, xeropthalmia, honey, Manuka honey, Royal Jelly, Leptospermum, Apis mellifera, management, therapy, Ocular Surface Disease Index; Tear breakup time test; Schirmer I test, corneal staining, aqueous-deficient dry eye disease, evaporative dry eye disease, lacrimal deficiency, lacrimal gland duct obstruction, drug-induced dry eye disease, vitamin A deficiency associated dry eye disease, contact lens wear associated dry eye disease, meibomian gland dysfunction.

### 4.3. Selection and Data Collection

Two authors (F.M. and J.P.) independently performed the database search. All the resulting titles were screened and, if suitable, the abstract of the articles was accessed. The full text of the abstracts which matched the topic of interest was accessed. A cross reference of the bibliography of the full-text articles was also screened for inclusion. Any disagreements were resolved by discussion, and a third author (N.M.) was involved in the final decision.

### 4.4. Data Items

Two authors (F.M. and J.P.) independently performed data extraction. The following data were extracted at baseline and at the last follow-up: Ocular Surface Disease Index [66], tear breakup time test [67], and Schirmer I test [68]. The primary outcome of interest was to investigate whether topical or oral honey-related treatment strategies improve the clinical outcome at the last follow-up compared to the baseline. The secondary outcome of interest was to compare honey-related therapy with placebo or artificial tears.

### 4.5. Study Risk of Bias Assessment

The between-studies risk of bias assessment was performed using the risk of bias tool of the Review Manager software (The Nordic Cochrane Collaboration, Copenhagen, Denmark). The following biases were evaluated by an independent author (J.P.): selection, performance, detection, attrition, reporting, and other sources of bias. The overall risk of publication bias was evaluated through the funnel plot. Asymmetry of the funnel plot is associated with a greater risk of publication bias.

### 4.6. Synthesis Methods

The statistical analysis was performed by the senior author (F.M.). To assess the improvement from the baseline to the last follow-up, the IBM SPSS software version 25 was used. Mean difference (MD), standard error (SE), T value and *t*-test were evaluated. For the comparisons, a meta-analysis was conducted using the Review Manager software (The Nordic Cochrane Collaboration, Copenhagen, Denmark) version 5.3. Data were analysed using the inverse variance and mean difference (MD) effect measure. The comparisons were performed with a fixed model effect as set-up. Heterogeneity was assessed through the Higgins-I^2^ test. If the I^2^ test was >50%, a random model effect was adopted. The confidence intervals (CI) were set at 95% in all analyses. Values of *p* < 0.05 were considered statistically significant. Forest plots were performed for each comparison.

## 5. Conclusions

According to the main findings of the present study, honey-related treatment strategies are an effective and feasible treatment option to improve symptoms and signs in patients with DED. Honey-related treatment strategies led to a significant increase in the tear breakup time and in the Schirmer I test and a significant reduction in the Ocular Surface Disease Index and corneal staining at the last follow-up. No significant differences were found in the tear breakup time, the Schirmer I test, corneal staining, or Ocular Surface Disease Index scores between the honey-related treatment and the control groups. No severe adverse effects were reported within the included studies. Future high-quality studies are needed to provide further evidence and to analyse the efficacy of different varieties of honey in the treatment of DED. Level I evidence studies are warranted to investigate the role of topical honey treatment in DED more extensively. In addition, future studies should determine which cohorts of DED patients could benefit from honey-related treatment options compared to other treatment options for DED, such as artificial tears or anti-inflammatory agents.

## Figures and Tables

**Table 1:** Generalities and patients baseline of the included study.

Author, Year	Journal	Follow-Up (Weeks)	Treatment	Patients (*n*)	Mean Age	Women (%)
Albietz et al., 2017 [36]	Clin. Exp. Optom.	8	Honey (Optimel Manuka gel) plus conventional therapy	37	58.9	42.9
			Honey (Optimel Manuka drops) plus conventional therapy	37	62.2	42.4
			Conventional therapy	40	61.4	41.2
Craig et al., 2020 [37]	Ocul. Surf.	13	Honey (Manuka microemulsion)	53	60.0	60.0
			No treatment	53	60.0	60.0
Inoue et al., 2017 [29]	PLoS ONE	8	Honey (Royal Jelly)	22	29.6	28.6
			Placebo	19	37.0	54.5
Tan et al., 2020 [38]	Br. J. Ophthalmol.	4	Honey (Optimel Manuka+ honey eye drops)	21	22.2	57.1
			Artificial tears	21	20.6	76.2
Wong et al., 2017 [27]	Cont. Lens Anterior Eye	2	Honey (Optimel Manuka drops)	10	25.7	55.0
			Artificial tears	10	25.7	55.0

**Table 2:** Comparison of the tear breakup time (s), the Ocular Surface Disease Index (points), the Schirmer I test (mm), and corneal staining (points) from baseline to the last follow-up (FU: follow-up; MD: mean difference; SE: standard error; 95% CI: 95% confidence interval).

Endpoint	Baseline	Last FU	MD	SE	95% CI	T-Value	*p*
Tear breakup time	5.0 ± 3.3	6.1 ± 2.7	1.1	0.426	0.25 to 1.94	2.58	0.01
Ocular Surface Disease Index	32.9 ± 9.9	20.1 ± 6.5	−12.8	1.184	−15.13 to −10.46	−10.808	<0.0001
Schirmer I test	16.7 ± 4.3	18.5 ± 1.7	1.8	0.462	0.88 to 2.71	3.893	0.0001
Corneal Staining	2.3 ± 2.6	1.1 ± 0.9	−1.2	0.275	−1.74 to −0.65	−4.361	<0.0001

## Data Availability

Not applicable.
